# 2,3-Dihydro-1*H*-cyclopenta[*b*]quinoline Derivatives as Acetylcholinesterase Inhibitors—Synthesis, Radiolabeling and Biodistribution

**DOI:** 10.3390/ijms130810067

**Published:** 2012-08-13

**Authors:** Paweł Szymański, Alice Lázničková, Milan Lázniček, Marek Bajda, Barbara Malawska, Magdalena Markowicz, Elżbieta Mikiciuk-Olasik

**Affiliations:** 1 Department of Pharmaceutical Chemistry and Drug Analyses, Medical University, ul. Muszyńskiego 1, Lodz 90-151, Poland; E-Mails: magdalena.markowicz@umed.lodz.pl (M.M.); elzbieta.mikiciuk-olasik@umed.lodz.pl (E.M.-O.); 2 Faculty of Pharmacy in Hradec Kralove, Charles University in Prague, Heyrovskeho 1203, Hradec Kralove CZ-50005, Czech Republic; E-Mails: alice.laznickova@faf.cuni.cz (A.L.); milan.laznicek@faf.cuni.cz (M.L.); 3 Department of Physicochemical Drug Analysis, Faculty of Pharmacy, Jagiellonian University Medical College, Medyczna 9, Krakow 30-688, Poland; E-Mails: marek.bajda@uj.edu.pl (M.B.); barbara.malawska@uj.edu.pl (B.M.)

**Keywords:** biological activity, medicinal chemistry, isotopic labeling, drug design, radiopharmaceuticals

## Abstract

In the present study we describe the synthesis and biological assessment of new tacrine analogs in the course of inhibition of acetylcholinesterase. The obtained molecules were synthesized in a condensation reaction between activated 6-BOC-hydrazinopyridine-3-carboxylic acid and 8-aminoalkyl derivatives of 2,3-dihydro-1*H*-cyclopenta[b]quinoline. Activities of the newly synthesized compounds were estimated by means of Ellman’s method. Compound **6h** (IC_50_ = 3.65 nM) was found to be most active. All obtained novel compounds present comparable activity to that of tacrine towards acetylcholinesterase (AChE) and, simultaneously, lower activity towards butyrylcholinesterase (BChE). Apart from **6a**, all synthesized compounds are characterized by a higher affinity for AChE and a lower affinity for BChE in comparison with tacrine. Among all obtained molecules, compound **6h** presented the highest selectivity towards inhibition of acetylcholinesterase. Molecular modeling showed that all compounds demonstrated a similar binding mode with AChE and interacted with catalytic and peripheral sites of AChE. Also, a biodistribution study of compound **6a** radiolabeled with ^99m^Tc was performed.

## 1. Introduction

Alzheimer’s disease (AD), the most common form of dementia with an incidence that is highly age dependent, is a degenerative disease of the central nervous system characterized by a noticeable cognitive decline. The most characteristic symptoms of AD are memory loss, deficit in learning ability, and a reduced ability to perform the basic activities of daily living. With the ageing of the world population, the prevalence, cost, and potential societal effects of AD will increase with certainty.

Several theories explaining the mechanism of AD development have been proposed by scientists over the past decades. Loss of cholinergic function, known as cholinergic hypothesis, amyloid cascade (amyloid hypothesis), oxidative stress, decrease of steroid hormone concentration, and an inflammatory process are most often mentioned [1]. The widely accepted cholinergic hypothesis has been verified repeatedly.

The most characteristic abnormality associated with AD is a decrease in central cholinergic neurotransmission, a consequence of decreased activity of choline acetyltransferase (ChAT), an enzyme that synthesizes acetylcholine (ACh) [2,3]. Acetylcholine, an ester of choline and acetic acid, acts in both the peripheral nervous system and central nervous system. The second enzyme which influences the level of ACh is acetylcholinesterase (AChE). In its natural state, it is a monomer with a molecular weight of approximately 60 kDa; often, however, it forms aggregates which continue to produce catalytic activity. The enzyme monomer is an α/β protein that contains 537 amino acids. It consists of a 12-stranded mixed beta sheet surrounded by 14 alpha helices [4].

Recent scientific reports suggest that AChE also plays a non-cholinergic role in the development of AD [5,6] by working as a chaperone molecule, accelerating the Aβ peptide deposition, and the aggregation of Aβ into insoluble fibrils [7,8].

One of the most popular therapeutic strategies in the treatment of AD is the control of cholinergic neurotransmission by slowing down the decline of neuronal degeneration or increasing cholinergic transmission [9]. Acetylcholinesterase inhibitors (AChEIs), which increase the synaptic amount of ACh by preventing its degradation, constitute the best-developed and widely approved class of AD drugs [10–13] and are used for mild-to-moderate stages of the disease. Results of clinical trials and imaging suggest that this group of drugs may lead also to a reduction in amyloid precursor protein (APP) formation [11]. Tacrine (1,2,3,4-tetrahydro-9-acridinamine-monohydrochloride) was the first drug approved by the United States Food and Drug Administration in 1993 for the palliative treatment of AD. It is a centrally active non-competitive reversible acetylcholinesterase inhibitor. However, its use is limited by a significant incidence of hepatotoxicity, cardiovascular system impairment, and mild cognitive benefits, while not altering the course of the disease [14].

Nevertheless, the search for new candidates–tacrine analogues is still of interest to scientists involved in AD research [15,16]. For example, 6-fluorotacrin-1-ol and 6-chlorotacrin-1-ol were found to be more potent than tacrine [17]. In the last decade, analogues containing two tacrine moieties linked by an alkylene chain were synthesized. It was reported that these dimeric molecules of tacrine were characterized by a stronger potency and a higher selectivity towards AChE [18,19]. It was proven that bis-(7)-tacrine had several modes of action, such as inhibition of AChE, *N-*Methyl*-*d*-*aspartate (NMDA) receptors, and nitric oxide synthase signaling. Furthermore, several homodimeric tacrine-based AChE inhibitors were synthesized. Their increased inhibitory potency was believed to be derived from the simultaneous binding of the units to the active and peripheral anionic sites of AChE [19]. One of these novel compounds, heptylene-linked bis-tacrine, was found to be 150-fold more active against rat AChE than tacrine and 250-fold more selective for acetylcholinesterase (AChE) than for butyrylcholinesterase (BChE) [20].

Second generation AChEIs (donepezil, rivastigmine and galantamine) demonstrate greater efficacy in AD treatment. Furthermore, when compared to tacrine, these drugs have fewer side effects and longer half-lives [6]. Therefore, synthesis of the analogues of the approved drugs: donepezil, rivastigmine, and galantamine is still of interest for many research groups [21,22].

Because of the complex pathophysiology of AD, involving numerous pathways, development of a satisfactory therapy is problematic. The key therapeutic targets are diffuse loss of neurons, reduced levels of the neurotransmitter acetylcholine (ACh), deposits of β-amyloid (Aβ) plaques, and neurofibrillary tangles [23]. For example, Tomassoli *et al*. reported that new 4-hydroxy-2-oxo-1,2-dihydroquinoline-3-carboxamides, 4-hydroxy-2-oxo-1,2-dihydroquinoline-3-carbohydrazide, and hexahydropyrimido [5,4-*c*]quinoline-2,5-diones were characterized by moderate activity towards AChE/BuChE inhibition; none of the synthesized compounds, however, showed higher inhibitory activity than tacrine [24]. Another scientific team investigated the potential activity of substituted 2-aminopyridine-3-carbonitriles towards AChE and BChE inhibition. Biological studies showed that some of these molecules were good AChE inhibitors, in the nanomolar range, and quite selective with regards to the inhibition of BChE [25].

The purpose of this study was to synthesize new derivatives of 2,3-dihydro-1*H*-cyclopenta [*b*]quinoline and nicotinic acid and to determine their activity towards inhibition of AChE and BChE. Furthermore, the selectivity of synthesized compounds was determined. This is significant for further phases of our study related to complexation of radioactive isotopes because during the design of novel potential radiopharmaceuticals selectivity and capability of binding with specified biological targets are more important than activity. Molecular modeling studies for synthesized compounds were also performed in order to elucidate the interactions between the enzymes and synthesized compounds. Furthermore, one of the synthesized molecules (compound **6a**) was labeled with ^99m^Tc and a biodistribution study of radioactivity was conducted following intravenous administration of ^99m^Tc-6a to rats in order to estimate its potential as diagnostic marker in AD.

## 2. Results and Discussion

### 2.1. Chemistry

**Scheme 1:**
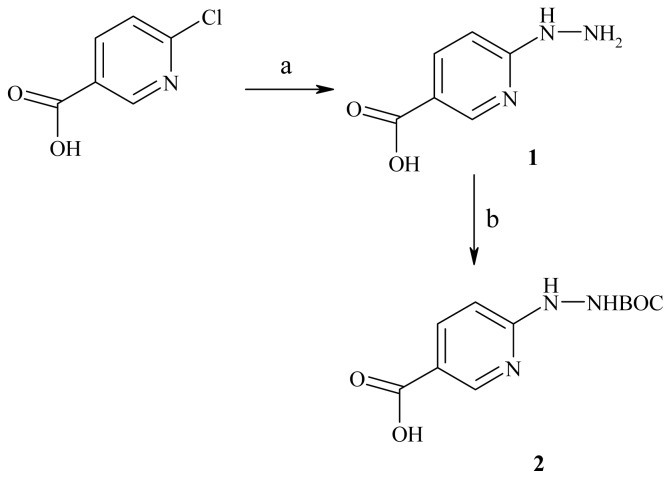
Synthesis of 6-BOC-hydrazinopyridine-3-carboxylic acid. Reagents: (**a**) 85% NH_2_NH_2_; (**b**) (*t*-BuOCO)_2_O, triethyl amine, DMF.

The first step of the synthesis was the preparation of 6-BOC-hydrazinopyridine-3-carboxylic acid (**2**). The substrate for this synthesis was 6-chloronicotinic acid. This compound reacted with hydrazine to give 6-hydrazinenicotinic acid (**1**). Subsequently, it was treated with di-*tert*-butyl dicarbonate and triethylamine in the presence of dimethylformamide (Scheme 1).

**Scheme 2:**
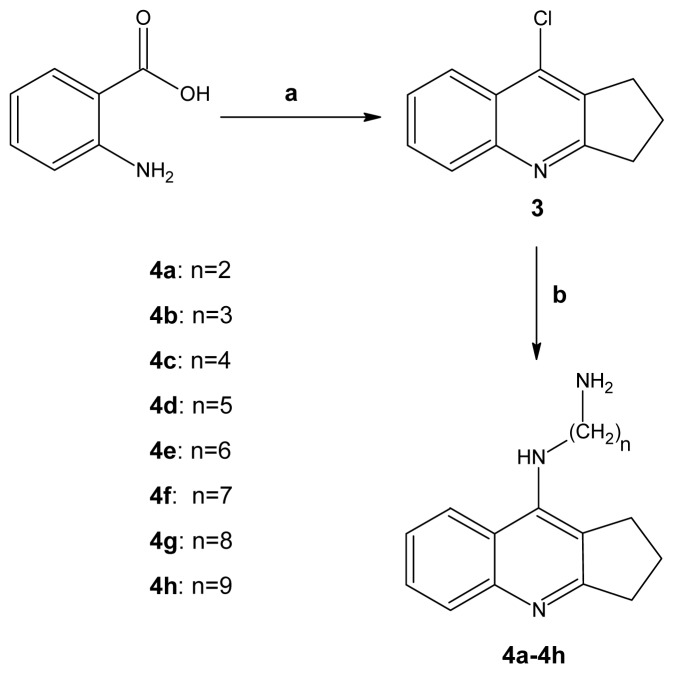
Synthesis of compounds (**3**), **4a**–**4h**. Reagents: (**a**) cyclopentanone, POCl_3_, reflux; (**b**) diamine, phenol, NaI, reflux.

These syntheses were described earlier by Abrams and co-workers [26]. 8-chloro-2,3-dihydro-1*H*-cyclopenta[*b*]quinoline (**3**) was prepared according to a procedure mentioned in a previous paper [17]. This reaction involved cyclization of anthranilic acid with cyclopentanone in POCl_3_. Subsequently, the obtained compound (**3**) was coupled with the appropriate alkyldiamine (number of carbon atoms ranging from 2 to 9) and giving 8-amino-2,3-dihydro-1*H*-cyclopenta[*b*]quinolines (Scheme 2) [27–31].

**Scheme 3:**
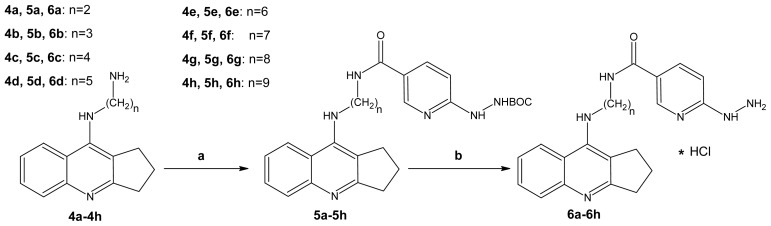
Synthesis of compounds **5a**–**5h** and **6a**–**6h**. Reagents: (**a**) Reactant 2, 2-chloro-4,6-dimethoxy-1,3,5-triazine (CDMT), *N*-methylmorpholine, THF; (**b**) HCl/ether.

Novel compounds were obtained via the synthesis between 6-(*N*′-*tert*butoxycarbonylhydrazino)-nicotinic acid (**2**), previously activated by 2-chloro-4,6-dimethoxy-1,3,5-triazine (CDMT), and *N*-methylmorpholine in solvent and reacted with compounds **4a**–**4h**. The best results (75% yield) in this reaction were achieved with drop by drop addition of *N*-methylmorpholine to the solution in tetrahydrofuran at −5 °C. Monitoring the reaction by TLC showed that the reaction of activation of the carboxylic group was usually completed within 1–4 h; subsequently, a mixture of the appropriate reactant **4a**–**4h** in the respective solvent was added at −5 °C. The last step of the synthesis involved conversion of the obtained compounds **5a**–**5h** into hydrochlorides **6a**–**6h** (Scheme 3) in the presence of hydrochloric acid with recrystallization from HCl in ether. In this step, the BOC group split off and the new compound precipitated.

### 2.2. Pharmacological Evaluation

#### 2.2.1. Studies of AChE/BChE Inhibition

The activity of the synthesized compounds towards inhibition of both enzymes (AChE and BChE) was estimated by Ellman’s spectrophotometric method [32–34]. Table 1 presents IC_50_ values of the newly obtained compounds with respect to AChE and BChE inhibition. Among these molecules, the most active towards inhibition of AChE appears to be molecule **6h** (IC_50_ = 3.65 nM). Compound **6g** (IC_50_ = 5.17 nM) exhibits a similar value of IC_50_ to tacrine. Obtained data shows that all synthesized molecules were characterized by lower BChE inhibitory activity in comparison with tacrine. Table 1 also lists values of relative inhibitory effects towards acetylcholinesterase (ratio IC_50_ BChE/AChE) and butyrylcholinesterase (ratio IC_50_ AChE/BChE). The most active molecule, compound **6h**, was more selective for AChE than tacrine. Derivative **6g**, with similar activity with regard to tacrine, was characterized by a higher selectivity for AChE in comparison with tacrine. All acquired compounds possessed lower affinity for BChE than tacrine; among all synthesized compounds, compound **6a** presented the highest activity for BChE. These findings are significant in view of the pathological processes involved in this type of neurodegenerative disease [35].

#### 2.2.2. Studies of Molecular Modeling

**Figure 1:**
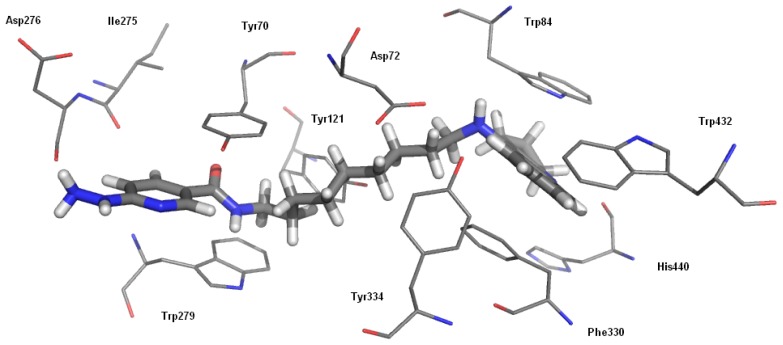
Binding mode of compound **6h** with acetylcholinesterase.

A novel series of compounds was docked to acetyl- and butyrylcholinesterase to show the possible interactions between inhibitors and enzymes. All ligands demonstrated a similar binding mode with AChE. They were extended along the active gorge and interacted with catalytic and peripheral sites. The most active molecule (compound **6h**) and its binding mode are presented in Figure 1. The fragment of the tacrine analogue with a cyclopentane ring created a characteristic sandwich due to p–p stacking with Trp84 and Phe330. The linker was located in the middle of the gorge, where it formed hydrophobic interactions with aromatic rings of Tyr121 and Tyr334. The nicotinamide moiety, in particular the amide bond, was located between two aromatic residues of the peripheral anionic site—Tyr70 and Trp279. Hydrazine interacted by H–bonding with the carbonyl group of the Asp276 backbone which was why the pyridine ring was shifted and was not able to create classical p–p stacking; it was engaged in some hydrophobic interactions.

**Figure 2:**
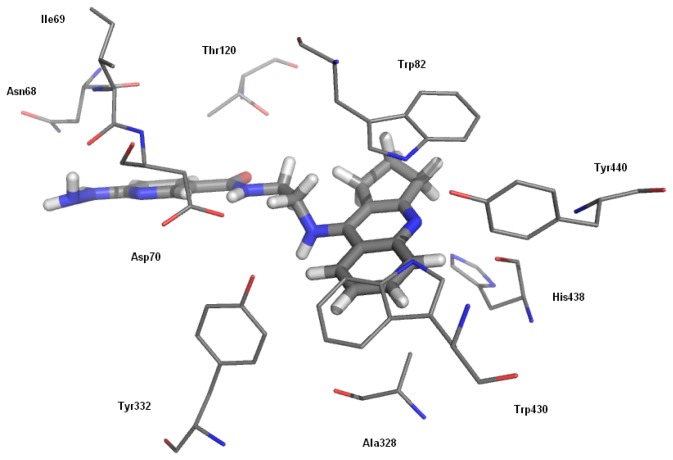
Binding mode of compound **6a** with butyrylcholinesterase.

In the case of butyrylcholinesterase, the binding mode of the cyclopentaquinoline moiety was similar. The small differences concerned the location of the hydrazinenicotinic fragment in the reduced peripheral anionic site of BChE. The binding mode of the most potent butyrylcholinesterase inhibitor **6a** is shown in Figure 2. A fragment of the tacrine analogue created p–p stacking with Trp82 and CH–p interactions with Trp430. The carbonyl group of the inhibitor amide bond formed H-bonding with OH Thr120 and the hydrazine moiety with the C=O of the Ile69 backbone.

#### 2.2.3. Radiolabeling with ^99m^Tc and Biodistribution Studies in Rats

**Figure 3:**
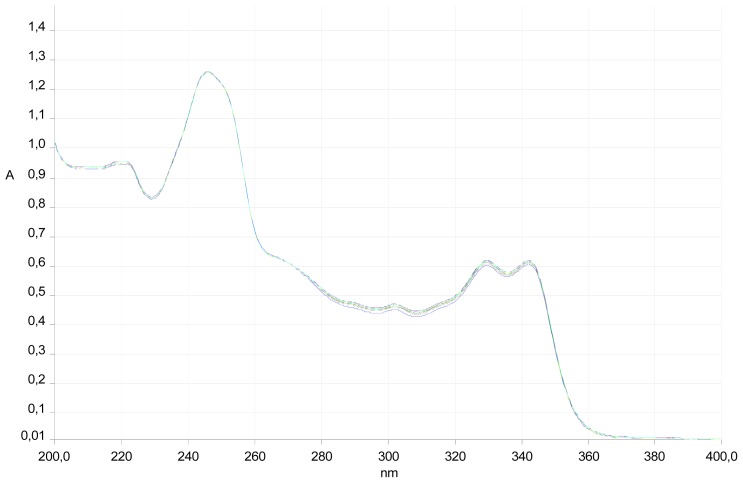
UV spectra of compound **6a** in aqueous solution at different times of incubation (0 to 4 h, measured every 15 min).

Compound **6a** was designed for radiolabeling. Also, spectrophotometric experiments were performed to determine its stability in water (Figure 3).

**Figure 4:**
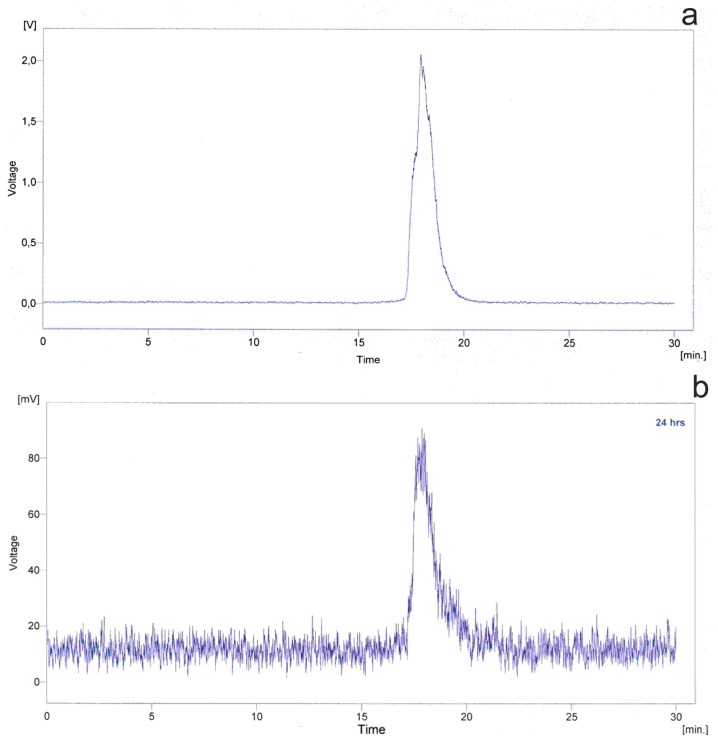
Radiochromatograms of complexes formed by technetium-99m with tricine and hydrazine nicotinate (HYNIC). (**a**) Compound **6a**; (**b**) Compound **6a** after 24 h.

Taking into consideration all synthesized compounds, compound **6a** is characterized by the highest selectivity towards BChE. Assessment of the level of this enzyme is very important as it is different in various stages of Alzheimer’s disease. Quality control of ^99m^Tc-labeled HYNIC-compounds with tricine as coligand with HPLC confirmed the purity of the product, which was found to be without unbound technetium (pertechnetate or hydrolyzed form, which would have appeared after a short elution time) [36]. Figure 4 presents radiochromatograms of complexes formed by technetium-99m with tricine and HYNIC.

Table 2 presents the tissue distribution of radioactivity after intravenous administration of ^99m^Tc-6a to rats. Collectively, compound **6a** exhibited relatively rapid blood radioactivity clearance; a large percentage of ^99m^Tc-radioactivity was located in the liver, but also partly in the kidney, lung, and the gastrointestinal tract. Whereas the liver radioactivity uptake persisted for a long time for ^99m^Tc-6a (Table 2), radioactivity found in the kidney and the gastrointestinal tract is more likely connected with elimination of the parent compounds and/or their metabolites from the body. Radioactivity concentrations in the brain were very low; probably, as a result of the hydrophilicity of radiolabelled compounds (the effect of technetium and co-ligands attached to the 2,3-dihydro-1*H*-cyclopenta [*b*]quinoline analogues), which are unable to cross the blood-brain barrier. This behavior is disadvantageous for the intended use of the agents for diagnosis of Alzheimer’s disease.

## 3. Experimental Section

### 3.1. Chemistry

During conducted syntheses dry organic solutions were used. This was achieved by employing anhydrous Na_2_SO_4_. Solvents were removed with a rotary evaporator. Melting points were determined by using an Electrothermal apparatus with open capillaries and were uncorrected. For monitoring of conducted reactions, TLC with 25 DC-Alufolien Kieselgel 60F_254_ (Merck) was used; detection was carried out with a UV Lamp (254 nm). Column chromatography was executed using silica gel 60 (200–400 mesh, Merck). For determining IR spectra, Mattson Infinity Series FT-IR spectrophotometer was used. IR spectra were recorded in KBr. ^1^H NMR spectra were recorded with Varian Mercury 300 MHz spectrometer, with tetramethylsilane as an internal standard. Mass spectra were performed by the Centre of Molecular and Macromolecular Studies in Lodz (Polish Academy of Sciences).

#### 3.1.1. 6-Hydrazinopyridine-3-carboxylic Acid (**1**)

6-Chloronicotinic acid (8.0 g, 50.77 mmol) was dissolved in 80% hydrazine hydrate (35 mL, 930.0 mmol) and placed in a 100 °C oil bath for 4 h. The homogeneous reaction mixture was cooled to room temperature and concentrated to dryness to give a white solid. The solid was dissolved in water and on acidification to pH 5.5 with concentrated hydrochloric acid, a precipitate was formed. The precipitate was isolated by filtration; the solid was washed with 95% ethanol and ether, and dried in vacuum. Compound **1**: yield 85%; mp 292–293 °C; ^1^H NMR (DMSO) (δ ppm.): 8.5 (1H, s, COOH), 8.3 (1H, s, CHN), 7.8 (1H, d, *J* = 2.4 Hz CCHC), 6.7 (1H, d, *J* = 8.8 Hz, CCHC), 3.2 (1H, s, CNH), 2.5 (2H, s, NH_2_); IR (KBr) ν (cm^−1^): 1333.0, 3080.1, 3308.2; MS (FAB) *m*/*z* (M + 1) 154.1; Elemental Analysis: Calc. for C_6_H_7_N_3_O_2_: C 47.06, H 4.61, N 27.44 Found: C 46.79 H 4.86 N 27.14.

#### 3.1.2. 6-BOC-hydrazinopyridine-3-carboxylic Acid (**2**)

To a solution of **1** (1.4 g, 9.8 mmol) and triethylamine (1.2 mL, 11.8 mmol) in DMF (10 mL) was added di-*tert*-butyl dicarbonate (2.13 g, 9.8 mmol). The reaction mixture became homogeneous over 1 h and stirring was continued for 16 h at room temperature. The reaction mixture was concentrated to dryness under reduced pressure to give a brown solid. Recrystallization from ethyl acetate gave the desired product **2** as a white solid. Compound **2**: yield 66%; mp 282–285 °C; ^1^H NMR (DMSO) (δ ppm.): 12.5 (1H, s, COOH), 8.9 (1H, d, *J* = 2.4 Hz NHC), 8.6 (1H, s, CHN), 7.9 (1H, d, *J* = 8.8 Hz, CCHC), 6.5 (1H, d, *J* = 8.8 Hz CCHC), 3.3 (1H, s, CNH), 1.9 (9H, s, BOC); IR (KBr) ν (cm^−1^): 1608.8, 1706.4, 3253.7; MS (FAB) *m*/*z* (M + 1) 254.2, 198.0; Elemental Analysis: Calc. for C_11_H_15_N_3_O_4_: C 52.17, H 5.97, N 16.59 Found: C 51.35, H 6.07, N 16.29.

#### 3.1.3. 9-Chloro-2,3-dihydro-1*H*-cyclopenta[*b*]quinoline (**3**)

To a mixture of anthranilic acid (7.4 g, 53.9 mmol) and cyclopentanone (1.64 mL, 53.9 mmol) was carefully added 30 mL of POCl_3_ in an ice bath. The mixture was heated under reflux for 2 h, then cooled to room temperature, and concentrated under reduced pressure to give a slurry. The residue was diluted with ethyl acetate (50 mL), neutralized with aqueous K_2_CO_3_ (30 mL), and washed with brine (2 × 20 mL). The organic layer was dried over MgSO_4_ and concentrated to dryness under reduced pressure to give a brown solid. Recrystallization from acetone gave the desired product **1** as a yellow solid. Compound **1**: yield 54%; mp 85–87 °C; ^1^H NMR (CDCl_3_) (δ ppm.): 8.1 (1H, d, *J* = 8.3 Hz, ArH), 7.9 (1H, d, *J* = 8.5 Hz, ArH), 7.6 (1H, t, *J* = 6.9 Hz, ArH), 7.4 (1H, t, *J* = 6.9 Hz, ArH), 3.0 (2H, d, *J* = 5.9 Hz, CH_2_), 2.9 (2H, d, *J* = 5.0 Hz, CH_2_), 1.8 (2H, t, *J* = 6.9 CH_2_); IR (KBr) ν (cm^−1^): 766.2, 1607.2, 2955.1, 2920.7, 3417.8.

#### 3.1.4. *N*′-(2,3-Dihydro-1*H*-cyclopenta[*b*]quinolin-9-yl)ethane-1,2-diamine (**4a**)

A mixture of **3** (0.71 g, 3.5 mmol), 1,2-diaminoethane (0.47 mL, 7 mmol), phenol (1.5 g), and NaI (0.07 g) was carefully heated at 180 °C for 2 h and then cooled to room temperature. The mixture was diluted with ethyl acetate (50 mL) and made basic with 10% KOH solution (30 mL). The organic layer was washed with water (20 mL) and brine (2 × 20 mL), and then dried over MgSO_4_ and concentrated under reduced pressure. The resulting residue was purified with silica gel chromatography (CH_2_Cl_2_: CH_3_OH:NH_3_ = 10:4.6:0.5) to afford **4a** as an oil. Compound **4a**: yield 64%; ^1^H NMR (CDCl_3_) (δ ppm.): 7.8 (1H, d, *J* = 8.3 Hz, ArH), 7.7 (1H, d, *J* = 8.3 Hz, ArH), 7.5 (1H, t, *J* = 6.9 Hz, ArH), 7.3 (1H, t, *J* = 8.1 Hz, ArH), 5.4 (1H, s, NH), 3.6 (2H, d, *J* = 5.4 Hz, NHCH_2_), 3.1 (2H, t, *J* = 7.3 Hz CH_2_NH_2_), 2.8–3.0 (4H, m, CH_2_), 2.6 (2H, p, *J* = 7.5, 7.5 Hz CH_2_), 1.6 (2H, s, NH_2_), IR (KBr) ν (cm^−1^): 1570.4, 2856.4, 2924.5, 2950.0, 3355.7; MS (FAB) *m*/*z* (M + 1) 228.1, 197.0, 185.0; MS-HR (FAB) Calc. for C_14_H_17_N_3_: 227.1422 Found: 227.14195.

#### 3.1.5. *N*′-(2,3-Dihydro-1*H*-cyclopenta[*b*]quinolin-9-yl)propane-1,3-diamine (**4b**)

A mixture of **3** (0.71 g, 3.5 mmol), 1,3-diaminopropane (0.58 mL, 7 mmol), phenol (1.5 g), and NaI (0.07 g) was combined as above to afford **4b** as an oil. Compound **4b**: yield 72%; ^1^H NMR (CD_3_OD) (δ ppm.): 8.0 (1H, d, *J* = 7.6 Hz, ArH), 7.7 (1H, d, *J* = 7.6 Hz, ArH), 7.6 (1H, t, *J* = 7.1 Hz, ArH), 7.4 (1H, t, *J* = 6.8 Hz, ArH), 3.7 (2H, t, *J* = 7.1 Hz, NHCH_2_), 3.3 (2H, t, *J* = 7.1 CH_2_NH_2_), 3.0 (2H, t, *J* = 7.1 Hz, CH_2_), 2.8 (2H, t, *J* = 7.1 Hz CH_2_), 2.1 (2H, p, *J* = 7.6, 7.8 Hz CH_2_), 1.9 (2H, p, *J* = 7.1, 7.1 Hz, CH_2_); IR (film) ν (cm^−1^): 1568.8, 2870.4, 2951.9, 3348.7; MS (FAB) *m*/*z* (M + 1) 242.2, 197.0, 185.0; MS-HR (FAB) Calc. for C_15_H_19_ N_3_: 241.1579 Found: 241.15789.

#### 3.1.6. *N*′-(2,3-Dihydro-1*H*-cyclopenta[*b*]quinolin-9-yl)butane-1,4-diamine (**4c**)

A mixture of **3** (0.71 g, 3.5 mmol), 1,6-diaminohexane (0.81 g, 7 mmol), phenol (1.5 g), and NaI (0.07 g) was combined as above to afford **4c** as an oil. Compound **4c**: yield 52%; ^1^H NMR (CD_3_OD) (δ ppm.): 8.0 (1H, d, *J* = 7.6 Hz, ArH), 7.7 (1H, d, *J* = 7.3 Hz, ArH), 7.5 (1H, t, *J* = 6.8 Hz, ArH), 7.4 (1H, t, *J* = 8.3 Hz, ArH), 3.6 (2H, t, *J* = 6.3 Hz, NHCH_2_), 3.2 (2H, t, *J* = 7.1 CH_2_NH_2_), 2.9 (2H, t, *J* = 7.8 Hz, CH_2_), 2.7 (2H, t, *J* = 7.1 Hz CH_2_), 2.1 (2H, p, *J* = 7.6, 7.6 Hz CH_2_), 1.6–1.8 (4H, m, CH_2_); IR (film) ν (cm^−1^): 1566.8, 2865.0, 2934.1, 3304.1; MS (FAB) *m*/*z* (M + 1) 256.2, 197.0, 185.0; MS-HR (FAB) Calc. for C_16_H_21_N_3_: 255.1735 Found: 255.17328.

#### 3.1.7. *N*′-(2,3-Dihydro-1*H*-cyclopenta[*b*]quinolin-9-yl)penthane-1,5-diamine (**4d**)

A mixture of **3** (0.71 g, 3.5 mmol), 1,5-diaminopentane (0.82 mL, 7 mmol), phenol (1.5 g), and NaI (0.07 g) was combined as above to afford **4d** as an oil. Compound **4d**: yield 75%; ^1^H NMR (CDCl_3_) (δ ppm.): 7.8 (1H, d, *J* = 7.3 Hz, ArH), 7.6 (1H, d, *J* = 7.5 Hz, ArH), 7.5 (1H, t, *J* = 6.9 Hz, ArH), 7.3 (1H, t, *J* = 6.9 Hz, ArH), 4.6 (1H, s, NH), 3.5 (2H, m, NHCH_2_), 3.1 (2H, t, *J* = 6.9 Hz, CH_2_), 3.0 (2H, t, *J* = 7.7 Hz, CH_2_NH_2_), 2.6 (2H, t, *J* = 6.7 Hz, CH_2_), 2.1 (2H, p, *J* = 3.9, 3.8 Hz, CH_2_), 1.9 (2H, s, NH_2_), 1.6 (2H, p, *J* = 7.3, 6.5 Hz, CH_2_), 1.3–1.5 (4H, br, CH_2_); IR (film) ν (cm^−1^): 1567.9, 2856.5, 2931.8, 3310.4; MS (FAB) *m*/*z* (M + 1) 270.3, 197.1, 185.0; MS-HR (FAB) Calc. for C_17_H_23_N_3_: 269.1892 Found: 270.19712 (M + 1).

#### 3.1.8. *N*′-(2,3-Dihydro-1*H*-cyclopenta[*b*]quinolin-9-yl)hexane-1,6-diamine (**4e**)

A mixture of **3** (0.71 g, 3.5 mmol), 1,6-diaminohexane (0.82 g, 7 mmol), phenol (1.5 g), and NaI (0.07 g) was combined as above to afford **4e** as an oil. Compound **4e**: yield 64%; ^1^H NMR (CD_3_OD) (δ ppm.): 8.0 (1H, d, *J* = 8.3 Hz, ArH), 7.7 (1H, d, *J* = 8.3 Hz, ArH), 7.5 (1H, t, *J* = 7.8 Hz, ArH), 7.3 (1H, t, *J* = 7.8 Hz, ArH), 3.6 (2H, t, *J* = 7.1 Hz, NHCH_2_), 3.2 (2H, t, *J* = 7.1 Hz, CH_2_), 3.0 (4H, t, *J* = 7.8 Hz, CH_2_NH_2_), 2.6 (2H, t, *J* = 6.8 Hz, CH_2_), 2.1 (2H, p, *J* = 7.6, 7.3 Hz, CH_2_), 1.6–1.7 (2H, m, CH_2_), 1.3–1.5 (6H, br, CH_2_); IR (film) ν (cm^−1^): 1567.5, 2855.0, 2928.4, 3350.4; MS (FAB) *m*/*z* (M + 1) 284.3, 197.0, 185.0; MS-HR (FAB) Calc. for C_18_H_25_N_3_: 283.2048 Found: 283.20426.

#### 3.1.9. *N*′-(2,3-Dihydro-1*H*-cyclopenta[*b*]quinolin-9-yl)heptane-1,7-diamine (**4f**)

A mixture of **3** (0.75 g, 3.5 mmol), 1,7-diaminoheptane (0.92 g, 7 mmol), phenol (1.5 g), and NaI (0.07 g) was combined as above to afford **4f** as an oil. Compound **4f**: yield 72%; ^1^H NMR (CDCl_3_) (δ ppm.): 7.9 (1H, d, *J* = 7.5 Hz, ArH), 7.7 (1H, t, *J* = 8.3 Hz, ArH), 7.5 (1H, t, *J* = 7.1 Hz, ArH), 7.4 (1H, t, *J* = 6.9 Hz, ArH), 4.6 (1H, s, NH), 3.6 (2H, m, NHCH_2_), 3.3 (2H, t, *J* = 7.5 Hz, CH_2_), 3.0 (2H, t, *J* = 7.7 Hz, CH_2_NH_2_), 2.6 (2H, t, *J* = 6.7 Hz, CH_2_), 2.1 (2H, p, *J* = 7.7, 7.3 Hz, CH_2_), 1.7 (2H, s, NH_2_), 1.5–1.6 (2H, m, CH_2_CH_2_), 1.2–1.5 (8H, br, CH_2_CH_2_); IR (film) ν (cm^−1^): 1567.9, 2853.9, 2927.9, 3294.4; MS (FAB) *m*/*z* (M + 1) 298.3, 197.0, 185.0; MS-HR (FAB) Calc. for C_19_H_27_N_3_: 297.2205 Found: 298.22911 (M + 1).

#### 3.1.10. *N*′-(2,3-Dihydro-1*H*-cyclopenta[*b*]quinolin-9-yl)octane-1,8-diamine (**4g**)

A mixture of **3** (0.71 g, 3.5 mmol), 1,8-diaminooctane (1.00 g, 7 mmol), phenol (1.5 g), and NaI (0.07 g) was combined as above to afford **4g** as an oil. Compound **4g**: yield 68%; ^1^H NMR (CDCl_3_) (δ ppm.): 7.8 (1H, d, *J* = 8.3 Hz, ArH), 7.5 (1H, t, *J* = 7.3 Hz, ArH), 7.3 (1H, t, *J* = 7.5 Hz, ArH), 4.5 (1H, s, NH), 3.5 (2H, m, NHCH_2_), 3.1 (2H, t, *J* = 7.1 Hz, CH_2_), 3.0 (2H, d, *J* = 7.7 Hz, CH_2_NH_2_), 2.6 (2H, t, *J* = 6.5 Hz, CH_2_), 1.5–1.6 (4H, br, CH_2_, NH_2_), 1.1–1.5 (10H, br, CH_2_); IR (film) ν (cm^−1^): 1567.2, 2853.2, 2925.5, 3329.5; MS (FAB) *m*/*z* (M + 1) 312.5, 197.1, 185.1; MS-HR (FAB) Calcd. for C_20_H_29_N_3_: 311.2361 Found: 312.24442 (M + 1).

#### 3.1.11. *N*′-(2,3-Dihydro-1*H*-cyclopenta[*b*]quinolin-9-yl)nonane-1,9-diamine (**4f**)

A mixture of **3** (0.71 g, 3.5 mmol), 1,9-diaminononane (1.10 g, 7 mmol), phenol (1.5 g), and NaI (0.07 g) was combined as above to afford **4f** as an oil. Compound **4f**: yield 66%; ^1^H NMR (CDCl_3_) (δ ppm.): 7.8 (1H, d, *J* = 7.3 Hz, ArH), 7.6 (1H, d, *J* = 7.5 Hz, ArH), 7.5 (1H, t, *J* = 6.9 Hz, ArH), 7.2 (1H, t, *J* = 6.9 Hz, ArH), 4.6 (1H, s, NH), 3.5 (2H, m, NHCH_2_), 3.1 (2H, t, *J* = 7.1 Hz CH_2_), 3.0 (2H, t, *J* = 7.7 Hz, CH_2_NH_2_), 2.6 (2H, t, *J* = 6.9 Hz CH_2_), 2.1 (2H, p, *J* = 7.7, 7.7 Hz CH_2_), 1,7 (2H, s, NH_2_), 1.5–1.6 (2H, m, CH_2_CH_2_), 1.1–1.3 (14H, br, CH_2_CH_2_); IR (film) ν (cm^−1^): 1568.0, 2852.5, 2925.7, 3294.4; MS (FAB) *m*/*z* (M + 1) 326.3, 185.0; MS-HR (FAB) Calc. for C_21_H_31_N_3_: 325.2518 Found: 326.26086 (M + 1).

#### 3.1.12. *N*-{5-[2-(2,3-Dihydro-1*H*-cyclopenta[*b*]quinolin-9-ylamino)ethylcarbamoyl]pyridin-2-yl} hydrazinecarboxylic Acid *tert*-Butyl Ester (**5a**)

To the 2-chloro-4,6-dimethoxy-1,3,5-triazine (CDMT) (1.76 g, 10 mmol) and **2** (2.53 g, 10 mmol) in THF (10 mL), *N*-methylmorpholine (1.1 mL, 10 mmol) was added drop by drop at a rate sufficient to keep the temperature between −5 °C and 0 °C. Stirring was continued at 0 °C for 1–4 h until all CDMT was consumed. Subsequently, to the crude mixture obtained as described above, **4a** (2.27 g, 10 mmol) in THF (8 mL) at −5 °C to 0 °C was added. Stirring was continued at 0 °C for 2 h, and then for 12 h at room temperature. Precipitate formed and was isolated by filtration. Recrystallization from ethyl acetate afforded the desired product **5a** as a yellow solid. Compound **5a**: yield 68%; mp 200–202 °C; ^1^H NMR (CD_3_OD) (δ ppm.): 8.5 (1H, s, ArH),8.3 (1H, d, *J* = 8.3 Hz, ArH), 7.9 (1H, d, *J* = 11.5 Hz, ArH), 7.8 (1H, t, *J* = 7.1 Hz, ArH), 7.7 (1H, d, *J* = 8.5 Hz, ArH), 7.6 (1H, t, *J* = 7.1 Hz, ArH), 6.7 (1H, d, *J* = 8.5 Hz, ArH), 4.0 (2H, t, *J* = 5.6 Hz, CH_2_), 3.7 (2H, t, *J* = 6.0 Hz, CH_2_), 3.4 (2H, t, *J* = 6.6 Hz, CH_2_), 3.1 (2H, t, *J* = 7.8 Hz, CH_2_), 2.3 (2H, p, *J* = 7.6, 7.8 Hz, CH_2_), 1.3–1.5 (9H, m, BOC); IR (KBr) ν (cm^−1^): 1464.2, 1610.9, 1740.6, 2869.2, 2936.2, 3422.1; MS (FAB) *m*/*z* (M + 1) 463.4, 363.2; 183.1; MS-HR (FAB) Calc. for C_25_H_30_N_6_O_3_: 462.23794 Found: 463.24583 (M + 1).

#### 3.1.13. *N*-{5-[3-(2,3-Dihydro-1*H*-cyclopenta[*b*]quinolin-9-ylamino)propylcarbamoyl]pyridin-2-yl} hydrazinecarboxylic Acid *tert*-Butyl Ester (**5b**)

A mixture of 2-chloro-4,6-dimethoxy-1,3,5-triazine (CDMT) (1.76 g, 10 mmol), **2** (2.53 g, 10 mmol) in THF (10 mL), and *N*-methylmorpholine (1.1 mL, 10 mmol) and after 4 h **4b** (2.41 g, 10 mmol) in THF were combined as above to afford **5b** as yellow solid. Compound **5b**: yield 66%; mp 159–161 °C; ^1^H NMR (CD_3_OD) (δ ppm.): 8.5 (1H, s, ArH),8.3 (1H, d, *J* = 8.5 Hz, ArH), 7.9 (1H, d, *J* = 9.0 Hz, ArH), 7.8 (1H, t, *J* = 5.9 Hz, ArH), 7.7 (1H, d, *J* = 7.6 Hz, ArH), 7.6 (1H, t, *J* = 8.5 Hz, ArH), 6.7 (1H, d, *J* = 8.5 Hz, ArH), 3.8 (2H, t, *J* = 6.8 Hz, CH_2_), 3.7 (2H, t, *J* = 4.6 Hz, CH_2_), 3.5 (2H, t, *J* = 6.4 Hz, CH_2_), 3.3 (2H, m, NH), 3.1 (2H, t, *J* = 7.8 Hz, CH_2_), 2.5 (2H, m, NH), 2.2 (2H, p, *J* = 7.8, 7.6 Hz, CH_2_), 2.0 (2H, p, *J* = 7.6, 5.1 Hz, CH_2_), 1.3–1.5 (9H, m, BOC); IR (KBr) ν (cm^−1^): 1470.7, 1635.8, 1721.1, 2853.4, 2967.2, 3259.6; MS (FAB) *m*/*z* (M + 1) 477.3, 377.3, 183.0; MS-HR (FAB) Calc. for C_26_H_32_N_6_O_3_: 476.25359 Found: 477.25943 (M + 1).

#### 3.1.14. *N*-{5-[4-(2,3-Dihydro-1*H*-cyclopenta[*b*]quinolin-9-ylamino)butylcarbamoyl]pyridin-2-yl} hydrazinecarboxylic Acid *tert*-Butyl Ester (**5c**)

A mixture of 2-chloro-4,6-dimethoxy-1,3,5-triazine (CDMT) (1.76 g, 10 mmol), **2** (2.53 g, 10 mmol) in THF (10 mL), and *N*-methylmorpholine (1.1 mL, 10 mmol) and after 4 h **4c** (2.55 g, 10 mmol) in THF were combined as above to afford **5c** as yellow solid. Compound **5c**: yield 67%; mp 145–148 °C; ^1^H NMR (CD_3_OD) (δ ppm.): 8.5 (1H, s, ArH),8.3 (1H, d, *J* = 7.9 Hz, ArH), 7.9 (1H, m, ArH), 7.8 (1H, t, *J* = 7.3 Hz, ArH), 7.7 (1H, d, *J* = 6.9 Hz, ArH), 7.6 (1H, t, *J* = 6.9 Hz, ArH), 6.7 (1H, d, *J* = 8.3 Hz, ArH), 3.8 (2H, t, *J* = 6.9 Hz, CH_2_), 3.7 (2H, t, *J* = 4.6 Hz, CH_2_), 3.3–3.4 (4H, m, NH, CH_2_), 3.1 (2H, t, *J* = 7.9 Hz, CH_2_), 2.5 (2H, m, NH), 2.3 (2H, p, *J* = 7.7, 9.3 Hz, CH_2_), 1.7 (4H, m, CH_2_), 1.3–1.5 (9H, m, BOC); IR (KBr) ν (cm^−1^): 1466.6, 1634.1, 1721.1, 2934.4, 3260.6; MS (FAB) *m*/*z* (M + 1) 491.3, 391.3, 185.1; MS-HR (FAB) Calc. for C_27_H_34_N_6_O_3_: 490.26924 Found: 491.27811 (M + 1).

#### 3.1.15. *N*-{5-[5-(2,3-Dihydro-1*H*-cyclopenta[*b*]quinolin-9-ylamino)pentylcarbamoyl]pyridin-2-yl} hydrazinecarboxylic Acid *tert*-Butyl Ester (**5d**)

A mixture of 2-chloro-4,6-dimethoxy-1,3,5-triazine (CDMT) (1.76 g, 10 mmol), **2** (2.53 g, 10 mmol) in THF (10 mL), and *N*-methylmorpholine (1.1 mL, 10 mmol) and after 4 h **4d** (2.69 g, 10 mmol) in THF were combined as above to afford **5d** as yellow solid. Compound **5d**: yield 70%; mp 106–108 °C; ^1^H NMR (CD_3_OD) (δ ppm.): 8.4 (1H, s, ArH),8.2 (1H, d, *J* = 8.7 Hz, ArH), 7.9 (1H, d, *J* = 8.9 Hz, ArH), 7.8 (1H, t, *J* = 5.6 Hz, ArH), 7.7 (1H, d, *J* = 8.1 Hz, ArH), 7.5 (1H, t, *J* = 8.3 Hz, ArH), 6.6 (1H, d, *J* = 8.9 Hz, ArH), 3.8 (2H, t, *J* = 6.9 Hz, CH_2_), 3.7 (2H, t, *J* = 4.8 Hz, CH_2_), 3.4 (2H, m, NH), 3.3 (2H, t, *J* = 7.1 Hz, CH_2_), 3.1 (2H, t, *J* = 7.7 Hz, CH_2_), 2.5 (2H, m, NH), 2.2 (2H, p, *J* = 7.9, 7.5 Hz, CH_2_), 1.6–1.8 (6H, m, CH_2_), 1.3–1.5 (9H, m, BOC); IR (KBr) ν (cm^−1^): 1465.2, 1634.4, 1716.0, 2855.0, 2927.0, 3275.6; MS (FAB) *m*/*z* (M + 1) 505.4, 405.3, 185.0; MS-HR (FAB) Calc. for C_28_H_36_N_6_O_3_: 504.28489 Found: 505.29257 (M + 1).

#### 3.1.16. *N*-{5-[6-(2,3-Dihydro-1*H*-cyclopenta[*b*]quinolin-9-ylamino)hexylcarbamoyl]pyridin-2-yl} hydrazinecarboxylic Acid *tert*-Butyl Ester (**5e**)

A mixture of 2-chloro-4,6-dimethoxy-1,3,5-triazine (CDMT) (1.76 g, 10 mmol), **2** (2.53 g, 10 mmol) in THF (10 mL), and *N-*methylmorpholine (1.1 mL, 10 mmol) and after 4 h **4c** (2.83 g, 10 mmol) in THF were combined as above to afford **5c** as yellow solid. Compound **5c**: yield 75%; mp 110–112 °C; ^1^H NMR (CD_3_OD) (δ ppm.): 8.5 (1H, s, ArH), 8.3 (1H, d, *J* = 8.1 Hz, ArH), 8.0 (1H, d, *J* = 9.1 Hz, ArH), 7.8 (1H, t, *J* = 5.7 Hz, ArH), 7.7 (1H, d, *J* = 7.5 Hz, ArH), 7.6 (1H, t, *J* = 7.1 Hz, ArH), 6.7 (1H, d, *J* = 8.9 Hz, ArH), 3.8 (2H, t, *J* = 7.2 Hz, CH_2_), 3.7 (2H, t, *J* = 4.8 Hz, CH_2_), 3.4 (4H, m, NH, CH_2_), 3.2 (2H, t, *J* = 5.4 Hz, CH_2_), 2.5 (2H, m, NH), 2.3 (2H, p, *J* = 7.7, 7.7 Hz, CH_2_), 1.8 (4H, m, CH_2_), 1.7 (4H, m, CH_2_), 1.3–1.5 (9H, m, BOC); IR (KBr) ν (cm^−1^): 1466.2, 1634.4, 1717.01, 2855.5, 2930.2, 3250.3; MS (FAB) *m*/*z* (M + 1) 519.3, 419.3, 185.0; MS-HR (FAB) Calc. for C_29_H_38_N_6_O_3_: 518.30054 Found: 519.30705 (M + 1).

#### 3.1.17. *N*-{5-[7-(2,3-Dihydro-1*H*-cyclopenta[*b*]quinolin-9-ylamino)heptylcarbamoyl]pyridin-2-yl} hydrazinecarboxylic Acid *tert*-Butyl Ester (**5f**)

A mixture of 2-chloro-4,6-dimethoxy-1,3,5-triazine (CDMT) (1.76 g, 10 mmol), **2** (2.53 g, 10 mmol) in THF (10 mL), and *N*-methylmorpholine (1.1 mL, 10 mmol) and after 4 h **4d** (2.97 g, 10 mmol) in THF were combined as above to afford **5d** as yellow solid. Compound **5d**: yield 66%; mp 105–107 °C; ^1^H NMR (CD_3_OD) (δ ppm.): 8.5 (1H, s, ArH), 8.3 (1H, d, *J* = 8.7 Hz, ArH), 8.0 (1H, d, *J* = 6.9 Hz, ArH), 7.8 (1H, t, *J* = 8.1 Hz, ArH), 7.7 (1H, d, *J* = 8.5 Hz, ArH), 7.6 (1H, t, *J* = 6.9 Hz, ArH), 6.7 (1H, d, *J* = 8.7 Hz, ArH), 4.0 (2H, t, *J* = 4.0 Hz, CH_2_), 3.8 (2H, t, *J* = 7.1 Hz, CH_2_), 3.4 (2H, t, *J* = 6.1 Hz, CH_2_), 3.2 (2H, t, *J* = 7.9 Hz, CH_2_), 2.3 (2H, p, *J* = 8.1, 6.9 Hz, CH_2_), 1.8 (4H, m, CH_2_), 1.6 (6H, m, CH_2_), 1.3–1.5 (9H, m, BOC); IR (KBr) ν (cm^−1^): 1566.5, 1717.7, 2855.1, 2927.2, 3295.0; MS (FAB) *m*/*z* (M + 1) 533.3, 433.3, 185.0; MS-HR (FAB) Calc. for C_30_H_40_N_6_O_3_: 532.31619 Found: 533.32329 (M + 1).

#### 3.1.18. *N*-{5-[8-(2,3-Dihydro-1*H*-cyclopenta[*b*]quinolin-9-ylamino)octylcarbamoyl]pyridin-2-yl} hydrazinecarboxylic Acid *tert*-Butyl Ester (**5g**)

A mixture of 2-chloro-4,6-dimethoxy-1,3,5-triazine (CDMT) (1.76 g, 10 mmol), **2** (2.53 g, 10 mmol) in THF (10 mL), and *N*-methylmorpholine (1.1 mL, 10 mmol) and after 4 h **4c** (3.11 g, 10 mmol) in THF were combined as above to afford **5c** as yellow solid. Compound **5c**: yield 46%; mp 134–136 °C; ^1^H NMR (CD_3_OD) (δ ppm.): 8.5 (1H, s, ArH), 8.3 (1H, d, *J* = 8.7 Hz, ArH), 8.0 (1H, d, *J* = 6.6 Hz, ArH), 7.8 (1H, t, *J* = 6.9 Hz, ArH), 7.7 (1H, d, *J* = 8.5 Hz, ArH), 7.6 (1H, t, *J* = 6.9 Hz, ArH), 6.7 (1H, d, *J* = 8.7 Hz, ArH), 4.6 (2H, m, NH), 4.0 (2H, t, *J* = 3.8 Hz, CH_2_), 3.8 (2H, t, *J* = 7.3 Hz, CH_2_), 3.4 (4H, m, NH,CH_2_), 3.2 (2H, t, *J* = 7.9 Hz, CH_2_), 2.3 (2H, p, *J* = 7.7, 6.9 Hz, CH_2_), 1.7 (6H, m, CH_2_), 1.6 (6H, m, CH_2_), 1.3–1.5 (9H, m, BOC); IR (KBr) ν (cm^−1^): 1466.7, 1634.1, 1717.2, 2854.3, 2928.2, 3259.6; MS (FAB) *m*/*z* (M + 1) 547.3, 447.3, 183.0; MS-HR (FAB) Calc. for C_31_H_42_N_6_O_3_: 546.33184 Found: 547.33977 (M + 1).

#### 3.1.19. *N*-{5-[9-(2,3-Dihydro-1*H*-cyclopenta[*b*]quinolin-9-ylamino)nonylcarbamoyl]pyridin-2-yl} hydrazinecarboxylic Acid *tert*-Butyl Ester (**5h**)

A mixture of 2-chloro-4,6-dimethoxy-1,3,5-triazine (CDMT) (1.76 g, 10 mmol), **2** (2.53 g, 10 mmol) in THF (10 mL), and *N*-methylmorpholine (1.1 mL, 10 mmol) and after 4 h **4d** (3.26 g, 10 mmol) in THF were combined as above to afford **5d** as yellow solid. Compound **5d**: yield 62%; mp 81–83 °C; ^1^H NMR (CD_3_OD) (δ ppm.): 8.5 (1H, s, ArH), 8.4 (1H, d, *J* = 8.3 Hz, ArH), 8.0 (1H, m, ArH), 7.8 (1H, t, *J* = 8.3 Hz, ArH), 7.7 (1H, d, *J* = 8.1 Hz, ArH), 7.5 (1H, t, *J* = 7.3 Hz, ArH), 6.6 (1H, d, *J* = 9.1 Hz, ArH), 4.6 (2H, m, NH), 4.0 (2H, m, CH_2_), 3.8 (2H, m, CH_2_), 3.4 (2H, m, NH), 3.0 (2H, m, CH_2_), 2.7 (2H, m, CH_2_), 2.0 (2H, p, *J* = 7.7, 6.9 Hz, CH_2_), 1.8 (6H, m, CH_2_), 1.7 (8H, m, CH_2_), 1.3–1.5 (9H, m, BOC); IR (KBr) ν (cm^−1^): 1489.8, 1636.3, 1709.1, 2868.7, 2927.8, 3421.7; MS (FAB) *m*/*z* (M + 1) 561.5, 461.3, 183.0; MS-HR (FAB) Calc. for C_32_H_44_N_6_O_3_: 560.34749 Found: 561.35526 (M + 1).

#### 3.1.20. 6-Hydrazino-*N*-[2-(2,3-dihydro-1*H*-cyclopenta[*b*]quinolin-9-ylamino)Ethyl]nicotinamide hydrochloride (**6a**)

Compound **5a** (0.20 g, 0.43 mmol) was dissolved in ether (2 mL), HCl/ether (4 mL) was added, and the reaction mixture was stirred at room temperature. After 24 h, the solution became cloudy and precipitate formed. The precipitate was isolated by filtration and the solid was washed with ether and dried. Compound **6a**: yield 34%; mp 235–136 °C; ^1^H NMR (CD_3_OD) (δ ppm.): 8.5 (1H, s, ArH), 8.4 (1H, d, *J* = 8.3 Hz, ArH), 8.2 (1H, d, *J* = 9.0 Hz, ArH), 7.9 (1H, t, *J* = 7.6 Hz, ArH), 7.7 (1H, d, *J* = 8.3 Hz, ArH), 7.6 (1H, t, *J* = 8.1 Hz, ArH), 7.0 (1H, d, *J* = 9.0 Hz, ArH), 4.0 (2H, t, *J* = 5.9 Hz, CH_2_), 3.8 (2H, t, *J* = 5.9 Hz, CH_2_), 3.5 (2H, t, *J* = 7.1 Hz, CH_2_), 3.1 (2H, t, *J* = 7.8 Hz, CH_2_), 2.3 (2H, p, *J* = 7.6, 7.6 Hz, CH_2_), IR (KBr) ν (cm^−1^): 1585.7, 1646.1, 2852.8, 2928.0, 3435.6; MS (FAB) *m*/*z* (M + 1) 363.2, 348.2; 185.0; MS-HR (FAB) Calc. for C_20_H_22_N_6_O: 362.18551 Found: 363.19414 (M + 1).

#### 3.1.21. 6-Hydrazino-*N*-[3-(2,3-dihydro-1*H*-cyclopenta[*b*]quinolin-9-ylamino)propyl]nicotinamide hydrochloride (**6b**)

A **5b** (0.20 g, 0.42 mmol) were combined as above to afford **6b** as brown solid. Compound **6b**: yield 36%; mp 226–229 °C; ^1^H NMR (DMSO) (δ ppm.): 14.1 (1H, s, HCl), 9.9 (1H, s, NH), 8.9 (1H, m, ArH), 8.8 (1H, m, ArH), 8.5 (1H, d, *J* = 8.7 Hz, ArH), 8.1 (1H, d, *J* = 8.9 Hz, ArH), 7.8 (1H, t, *J* = 7.3 Hz, ArH), 7.6 (1H, t, *J* = 7.7 Hz, ArH), 6.9 (1H, d, *J* = 8.5 Hz, ArH), 3.7 (4H, m, NH), 3.4–3.6 (4H, m, CH_2_), 3.3 (2H, t, *J* = 6.9 Hz, CH_2_), 3.1 (2H, t, *J* = 8.3 Hz, CH_2_), 2.1 (2H, m, Hz, CH_2_), 1.9 (2H, m, CH_2_); IR (KBr) ν (cm^−1^): 1585.3, 1648.5, 2857.0, 2933.0, 3277.6; MS (FAB) *m*/*z* (M + 1) 377.2, 362.1; 185.0 MS-HR (FAB) Calc. for C_21_H_24_N_6_O: 376.20116 Found: 377.21072 (M + 1).

#### 3.1.22. 6-Hydrazino-*N*-[4-(2,3-dihydro-1*H*-cyclopenta[*b*]quinolin-9-ylamino)butyl]nicotinamide hydrochloride (**6c**)

A **5c** (0.20 g, 0.41 mmol) were combined as above to afford **6c** as yellow solid. Compound **6c**: yield 30%; mp 171–173 °C; ^1^H NMR (DMSO) (δ ppm.):14.3 (1H, s, HCl), 9.9 (1H, s, NH), 8.8 (1H, m, ArH), 8.7 (1H, m, ArH), 8.6 (1H, m, ArH), 8.1 (1H, d, *J* = 8.9 Hz, ArH), 7.8 (1H, t, *J* = 8.3 Hz, ArH), 7.6 (1H, t, *J* = 6.7 Hz, ArH), 6.9 (1H, d, *J* = 8.5 Hz, ArH), 3.7 (4H, m, NH), 3.3–3.4 (4H, m, CH_2_), 3.2 (2H, t, *J* = 7.7 Hz, CH_2_), 3.1 (2H, t, *J* = 7.7 Hz, CH_2_), 2.1 (2H, m, CH_2_), 1.6–1.8 (4H, m, CH_2_); IR (KBr) ν (cm^−1^): 1541.7, 1647.7, 2857.4, 2904.7, 3414.7; MS (FAB) *m*/*z* (M + 1) 391.4, 376.4, 185.0; MS-HR (FAB) Calc. for C_22_H_26_N_6_O: 391.21681 Found: 391.22435 (M + 1).

#### 3.1.23. 6-Hydrazino-*N*-[5-(2,3-dihydro-1*H*-cyclopenta[*b*]quinolin-9-ylamino)pentyl]nicotinamide hydrochloride (**6d**)

A **5d** (0.20 g, 0.40 mmol) were combined as above to afford **6d** as brown solid. Compound **6d**: yield 36%; mp 107–108 °C; ^1^H NMR (DMSO) (δ ppm.):14.4 (1H, s, HCl), 10.0 (1H, s, NH), 8.9 (1H, m, ArH), 8.8 (2H, m, ArH), 8.1 (1H, d, *J* = 8.9 Hz, ArH), 7.8 (1H, t, *J* = 10.1 Hz, ArH), 7.6 (1H, t, *J* = 7.5 Hz, ArH), 6.9 (1H, d, *J* = 8.9 Hz, ArH), 3.7 (4H, m, NH), 3.3 -3.4 (4H, m, CH_2_), 3.2 (2H, t, *J* = 9.9 Hz, CH_2_), 3.1 (2H, t, *J* = 7.5 Hz, CH_2_), 2.1 (2H, p, *J* = 7.5, 7.1 Hz, CH_2_), 1.4–1.6 (6H, m, CH_2_); IR (KBr) ν (cm^−1^): 1558.4, 1647.7, 2858.4, 2931.2, 3385.2; MS (FAB) *m*/*z* (M + 1) 405.3, 390.3, 185.0; MS-HR (FAB) Calc. for C_23_H_28_N_6_O: 404.23246 Found: 405.24165 (M + 1).

#### 3.1.24. 6-Hydrazino-*N*-[6-(2,3-dihydro-1*H*-cyclopenta[*b*]quinolin-9-ylamino)hexyl]nicotinamide hydrochloride (**6e**)

A **5e** (0.20 g, 0.39 mmol) were combined as above to afford **6e** as yellow solid. Compound **6e**: yield 33%; mp 166–167 °C; ^1^H NMR (DMSO) (δ ppm.):14.4 (1H, s, HCl), 10.0 (1H, s, NH), 8.9 (1H, m, ArH), 8.8 (2H, m, ArH), 8.1 (1H, d, *J* = 6.7 Hz, ArH), 7.9 (1H, t, *J* = 8.1 Hz, ArH), 7.6 (1H, t, *J* = 8.1 Hz, ArH), 6.9 (1H, d, *J* = 8.9 Hz, ArH), 3.7 (4H, m, NH), 3.4–3.5 (4H, m, CH_2_), 3.3 (2H, t, *J* = 7.1 Hz, CH_2_), 3.1 (2H, t, *J* = 7.7 Hz, CH_2_), 2.2 (2H, p, *J* = 7.7, 7.1 Hz, CH_2_), 1.4–1.7 (8H, m, CH_2_); IR (KBr) ν (cm^−1^): 1559.3, 1636.9, 2857.4, 2930.2, 3424.8; MS (FAB) *m*/*z* (M + 1) 419.3, 404.3, 185.0; MS-HR (FAB) Calc. for C_24_H_30_N_6_O: 418.24811 Found: 419.25754 (M + 1).

#### 3.1.25. 6-Hydrazino-*N*-[7-(2,3-dihydro-1*H*-cyclopenta[*b*]quinolin-9-ylamino)heptyl]nicotinamide hydrochloride (**6f**)

A **5f** (0.20 g, 0.38 mmol) were combined as above to afford **6f** as brown solid. Compound **6f**: yield 43%; mp 105–107 °C; ^1^H NMR (DMSO) (δ ppm.):14.4 (1H, s, HCl), 10.0 (1H, s, NH), 8.8 (1H, m, ArH), 8.6 (2H, m, ArH), 8.1 (1H, d, *J* = 6.5 Hz, ArH), 7.9 (1H, t, *J* = 9.5 Hz, ArH), 7.6 (1H, t, *J* = 6.7 Hz, ArH), 6.9 (1H, d, *J* = 8.9 Hz, ArH), 3.7 (4H, m, NH), 3.4 (4H, m, CH_2_), 3.2 (2H, t, *J* = 7.5 Hz, CH_2_), 3.1 (2H, t, *J* = 7.7 Hz, CH_2_), 2.2 (2H, p, *J* = 7.1, 7.1 Hz, CH_2_), 1.3–1.7 (10H, m, CH_2_); IR (KBr) ν (cm^−1^): 1558.9, 1648.0, 2855.3, 2926.6, 3259.7, 3405.0; MS (FAB) *m*/*z* (M + 1) 433.3, 418.3, 185.0; MS-HR (FAB) Calc. for C_25_H_32_N_6_O: 432.26376 Found: 433.27207 (M + 1).

#### 3.1.26. 6-Hydrazino-*N*-[8-(2,3-dihydro-1*H*-cyclopenta[*b*]quinolin-9-ylamino)octyl]nicotinamide hydrochloride (**6g**)

A **5g** (0.20 g, 0.37 mmol) were combined as above to afford **6g** as yellow solid. Compound **6g**: yield 43%; mp 170–173 °C; ^1^H NMR (DMSO) (δ ppm.):14.2 (1H, s, HCl), 9.9 (1H, s, NH), 8.8 (1H, m, ArH), 8.6 (2H, m, ArH), 8.1 (1H, d, *J* = 8.9 Hz, ArH), 7.8 (1H, m, ArH), 7.6 (1H, m, ArH), 6.9 (1H, d, *J* = 8.3 Hz, ArH), 3.7 (4H, m, NH), 3.3 (4H, m, CH_2_), 3.2 (2H, t, *J* = 5.7 Hz, CH_2_), 3.1 (2H, t, *J* = 9.1 Hz, CH_2_), 2.2 (2H, p, *J* = 7.3, 7.3 Hz, CH_2_), 1.3–1.8 (12H, m, CH_2_); IR (KBr) ν (cm^−1^): 1558.5, 1634.0, 2854.2, 2926.7, 3200.6, 3422.2; MS (FAB) *m*/*z* (M + 1) 447.1, 432.2, 185.0; MS-HR (FAB) Calc. for C_26_H_34_N_6_O: 446.27941 Found: 447.28878 (M + 1).

#### 3.1.27. 6-Hydrazino-*N*-[9-(2,3-dihydro-1*H*-cyclopenta[*b*]quinolin-9-ylamino)nonyl]nicotinamide hydrochloride (**6h**)

A **5h** (0.20 g, 0.35 mmol) were combined as above to afford **6h** as brown solid. Compound **6h**: yield 44%; mp 85–87 °C; ^1^H NMR (DMSO) (δ ppm.):14.5 (1H, s, HCl), 10.0 (1H, s, NH), 8.9 (1H, m, ArH), 8.8 (2H, m, ArH), 8.1 (1H, d, *J* = 6.9 Hz, ArH), 7.8 (1H, t, *J* = 6.7 Hz, ArH), 7.6 (1H, t, *J* = 7.1 Hz, ArH), 6.9 (1H, d, *J* = 8.9 Hz, ArH), 3.7 (4H, m, NH), 3.4 (4H, m, CH_2_), 3.3 (2H, m, CH_2_), 3.2 (2H, t, *J* = 7.7 Hz, CH_2_), 2.1 (2H, m, CH_2_), 1.5–1.7 (14H, m, CH_2_); IR (KBr) ν (cm^−1^): 1585.8, 1647.3, 2853.5, 2925.4, 3438.1; MS (FAB) *m*/*z* (M + 1) 461.3, 446.3, 183.0; MS-HR (FAB) Calc. for C_27_H_36_N_6_O: 460.29506 Found: 461.30121 (M + 1).

### 3.2. Biochemical Studies

Determination of the inhibitory activity of all synthesized compounds towards AChE and BChE was performed by means of Ellman’s spectrophotometric method with our own modifications. Every sample contained 5,5′-dithiobisnitrobenzoic acid (DTNB, 0.05 mL, 0.5 M), acetylthiocholine iodide (substrate), newly synthesized inhibitor, and AChE (5 units/mL) or BChE (5 units/mL), respectively, in cholinesterases activity measurements. Every measurement was conducted in the presence of phosphate buffer (0.1 M, pH 8.0) at 37 °C. The total volume of every sample amounted to 3 mL. Measurement procedure was identical for all samples. All ingredients were incubated for 1 min and then the absorbance was recorded at 412 nm. Seven concentrations of acetylthiocholine iodide were used in order to obtain the inhibition curves for every compound. Every measurement was conducted three times. Also, samples without inhibitor were evaluated in order to obtain absolute AChE and BChE activity. The value of IC_50_, defined as the drug concentration that contributes to the inhibition of 50% AChE or BChE activity, was determined by non-linear and linear regression.

All reagents: DTNB, enzymes (C2629 Acetylcholinesterase from *Electrophorus electricus* (electric eel) and C4290 Butyrylcholinesterase from equine serum) and acetylthiocholine iodide were purchased from Sigma-Aldrich.

### 3.3. Molecular Modeling

The three-dimensional structures of inhibitors were created by Corina on-line (Molecular Networks) and subsequently prepared with Sybyl 8.0 (Tripos). Atom types were checked, hydrogen atoms were added, and, then, Gasteiger-Marsili charges were assigned. Ligands were bound to acetylcholinesterase from 2CKM and butyrylcholinesterase from 1P0I crystal complex. Protein was prepared before binding with GoldSuite 5.0.1 (CCDC). All histidine residues were protonated at Nɛ, the hydrogen atoms were added, ligand and water molecules were removed, and the binding site was defined as all amino acid residues within 10 Å from bis-(7)-tacrine for AChE and 20 Å from the glycerol molecule present in the active center of BChE. A standard set of genetic algorithms with population size of 100, number of operations 100,000, and clustering with a tolerance of 1Å was applied. As a result 10 ligand poses, sorted by GoldScore (AChE) and ChemScore (BChE) function value were obtained. The results were visualized by PyMOL 0.99rc6 (DeLano Scientific LLC).

### 3.4. Spectrophotometric Experiments

The absorption spectrum was obtained by scanning the sample between 200 and 350 nm with a Perkin Elmer spectrophotometer. The experiment was started with a solution of pure water at room temperature. The stability of compound **6a** was assessed by monitoring the variability of the spectrum at regular intervals (15 min) over 4 h.

### 3.5. Radiolabelling

For radiolabeling with Tc-99m, 1 mg of the ligand **6a** was dissolved in 150 μL of water. Subsequently, 100 mg of tricine and 1.5 mL of technetium eluate were added together with 25 μL of SnCl_2_ in ethanol (1 mg/mL). After 30 min incubation at room temperature, quality control was performed by HPLC analysis. HPLC analysis was performed on an Agilent System 1100 Series with UV and radiometric detection, with LiChrocart column 250-3 Luichrospher 100 RP-18 (5 μm). Flow rate was 1 mL/min.

Gradient I:

Mobile phase A: 0.9% NaCl, B: CH_3_CN.
1–25 min 50% B.
25–30 min 50%–100% B.
30–35 min 100% B.
35–40 min 100%–0% B.

Gradient II:

0–10 min 0% B.
10–25 min 0%–100% B.
25–30 min 100% B.
30–35 min 100%–0% B.

### 3.6. Biodistribution Studies in Rats

#### 3.6.1. Animals

For biological experiments, the radiolabelled compound was dissolved in saline to a concentration of the ligand equal to 100 μg/mL. Intravenous dose was 20 μg per animal

For biodistribution studies, male Wistar rats weighing 190–260 g were used. Prior to the experiment, the animals were fasted overnight (to empty the bowels), but had free access to water. All animal experiments were approved by the Ethics Committee of the Faculty of Pharmacy, Charles University, Hradec Kralove.

#### 3.6.2. Biodistribution in Rats

The agent was administered to rats intravenously in a volume of 0.2 mL. During the course of the experiments, each animal was placed in an individual cage. At various time points after injection, the carotid artery was exposed under ether anesthesia and a blood sample was collected in glass tubes containing dry heparin. The rats were sacrificed and dissected. The organs of interest were weighed and counted for radioactivity in an automatic gamma counter (1480 Wizard 3).

The results were expressed as mean ± standard deviations of at least four animals.

## 4. Conclusions

As a consequence of a limited number of efficacious drugs in the treatment of AD, many scientific teams are aiming to discover novel compounds able to improve cholinergic neurotransmission. Currently approved AChE inhibitors, such as galantamine, rivastigmine, and donepezil, provide comparatively little chance for a prolonged improvement in cognitive functions. Thus, the search for novel compounds with anticholinesterase activity continues as numerous scientists are focused on the development of novel compounds such as cystamine-tacrine dimers [37], bisquaternary isoquinolinium derivatives [38], diversely substituted furo[2,3-*b*]quinolin-4-amine and pyrrolo[2,3-*b*]quinolin-4-amine derivatives [39], and tacrine-8-hydroxyquinoline hybrid [40] that might increase the level of ACh.

Several teams have also utilized derivatives of tacrine as potential radiopharmaceuticals. Tacrine and its modified structure derivatives were marked with radioactive isotope in order to determine the level of AChE or BChE as a function of tacrine analogue labeled isotope accumulated in the target site [41,42]. Also, other AChE inhibitors such as donepezil and huperzine were modified and marked with radioisotopes [43,44].

In our previous papers, we presented the synthesis and biological evaluation of derivatives of tetrahydroacridine derivatives with hydrazine nicotinate (HYNIC) moiety [45,46]. The fragment of tetrahydroacridine that possesses the possibility to inhibit both the moieties of cholinesterase and HYNIC has the potential to be utilized as a co-ligand for radiolabeling.

As described in this article, eight synthesized compounds differed from each other only in the length of the aliphatic chain between the tetrahydroacridine and the hydrazine nicotinate moiety. According to the results of the studies, compounds showing the highest activity with regard to AChE inhibition were those with six and eight carbon atoms in the aliphatic chain; which were approximately 7- and 9- fold more active than tacrine, respectively. This data suggests that altering the molecule by incorporating a longer aliphatic chain proved to be a good choice. All novel compounds, apart from the one with six carbon atoms in the aliphatic chain, described in these studies were characterized, in comparison to tacrine, by lower inhibitory activity towards BChE [45,46].

Within this work, compared to our previous studies, the six membered ring of tetrahydroacridine was exchanged with a five membered ring; this influenced the activity and interaction with active sites. Of all the synthesized compounds, the most active was **6h** (IC_50_ = 3.65 nM). This compound was about 1.5-fold more active than tacrine and, as compared to this reference compound, more selective towards AChE. Compound **6g** exhibited a similar value of IC_50_ to that of tacrine. Similarly to our previous studies, obtained data suggests that the activity of the synthesized compounds increases simultaneously with the length of the aliphatic chain between the hydrazine nicotinate moiety and 2,3-dihydro-1*H*-cyclopenta[*b*]quinolone. Our data showed that all synthesized molecules were characterized by lower BChE inhibitory activity in comparison to tacrine.

On the other hand, selectivity of the obtained compounds is very promising because the moiety of 6-hydrazinenicotinic acid (HYNIC) is thought to be responsible for the binding of technetium-99m radiotracer (^99m^Tc) (as reported previously by Abrams *et al*.) [26].

According to molecular modeling studies, all ligands were extended along the active gorge and interacted with both the catalytic and peripheral site of AChE. This dual type of binding to AChE is responsible for an additional function related to the interaction with β-amyloid. The mode of binding with BChE was similar; the main difference was the location of the hydrazinenicotinic fragment in the reduced peripheral anionic site of BChE. These results are of vital importance as it has been established that AChE not only plays a crucial role in cholinergic dysfunction, but also is involved in the β-amyloid cascade via the AChE peripheral anionic site (PAS), such as mediating the adhesion, differentiation and deposition of β-amyloid in AD. It has been reported that AChE is a protein associated with the amyloid core of mature senile plaques, pre-amyloid diffuse deposits, and cerebral blood vessels in AD brain. Alvares *et al.*, in *in vitro* studies showed that AChE is incorporated into β-amyloid aggregates by forming macromolecular complexes with the growing β-amyloid fibrils. The following scientific work of Alvares demonstrated that these complexes accelerate the maturation of β-amyloid plaques and are more toxic at the cellular level than the amyloid fibrils alone [47,48]. Molecular modeling studies presented within this work revealed that synthesized compounds are dual binding site inhibitors; thus, there is a possibility to influence non-cholinergic functions of AChE including AChE-induced aggregation of β-amyloid.

Biodistribution studies in rats revealed that compound **6a** exhibited comparatively rapid blood radioactivity clearance. ^99m^Tc-radioactivity was mainly located in the liver, and to a lesser extent in the kidney, lung, and the gastrointestinal tract. Low radioactivity concentrations in the brain suggest that these agents do not cross the blood-brain barrier, and, therefore, cannot be regarded as potential agents for diagnosis of Alzheimer’s disease. However, similarly to [^11^C]choline, the synthesized compounds might be further evaluated as molecules suitable for the detection of cancers in certain organs (e.g., liver, kidney, lungs) or to monitor the response to various therapies. For example, there are scientific reports which prove that [^11^C]choline, developed by Hara *et al.*, as an oncologic PET (positron emission tomography) radiopharmaceutical, might be utilized with good results in the diagnosis of lung cancer [49], colon cancer [50], or prostate cancer [51]. Furthermore, [^11^C]choline and multimodality fusion imaging with integrated PET and contrast-enhanced CT (PET/CT) could be used to monitor the response to anti-androgenic therapy [52].

Results of our synthesis and analysis suggest that the obtained hybrids of 2,3-dihydro-1*H*-cyclopenta[*b*]quinolone and 6-hydrazinonicotynic acid may be considered as novel potential anti-Alzheimer’s drugs. Conversely, these compounds, following radiolabeling, could be used in the detection of the cholinergic deficit occurring in the peripheral nervous system or in the diagnosis of various types of cancers or to correct physiological functions.

## Figures and Tables

**Table 1:** IC_50_ values for activities towards acetylcholinesterase (AChE) and butyrylcholinesterase (BChE).

Compound	AChE IC_50_, (nM) ± SEM a	BChE IC_50_, (nM) ± SEM b	Selectivity for AChE c	Selectivity for BChE d
**6a**	31.50 ± 1.9	164.00 ± 1.6	5.21	0.19
**6b**	19.30 ± 1.1	197.00 ± 3.4	10.21	0.10
**6c**	22.40 ± 1.7	2650.00 ± 9.5	118.30	0.01
**6d**	7.84 ± 2.2	4240.00 ± 2.2	540.82	0.00
**6e**	41.60 ± 1.0	16600.00 ± 17.5	399.04	0.00
**6f**	17.60 ± 0.8	22700.00 ± 20.6	1289.77	0.00
**6g**	5.17 ± 1.4	19600.00 ± 16.4	3791.10	0.00
**6h**	3.65 ± 0.5	17100.00 ± 21.3	4684.93	0.00
**tacrine**	5.46 ± 1.0	2.44 ± 0.6	0.45	2.24

a Inhibitor concentration (means ± SEM of three experiments) for 50% inactivation of AChE;

b Inhibitor concentration (means ± SEM of three experiments) for 50% inactivation of BChE;

c Selectivity for AChE is defined as IC_50_(BChE)/IC_50_(AChE);

d Selectivity for BChE is defined as IC_50_(AChE)/IC_50_(BChE).

**Table 2:** Distribution of radioactivity in selected organs and systems of rats after intravenous administration of **^99m^Tc-6a**.

^99m^Tc-6a (% Dose/g)				

Organs	5 min	60 min	120 min	24 h
Blood	0.850 ± 0.108	0.114 ± 0.015	0.091 ± 0.017	0.033 ± 0.004
Plasma	0.921 ± 0.124	0.183 ± 0.024	0.150 ± 0.027	0.052 ± 0.003
Pancreas	0.551 ± 0.109	0.117 ± 0.019	0.092 ± 0.015	0.067 ± 0.006
Liver	5.752 ± 0.742	5.160 ± 0.586	5.630 ± 0.508	5.095 ± 0.478
Adrenals	1.186 ± 0.231	0.900 ± 0.102	0.890 ± 0.040	0.793 ± 0.137
Kidney	3.838 ± 0.264	1.556 ± 0.148	1.332 ± 0.209	1.026 ± 0.130
Lung	3.497 ± 0.239	2.010 ± 0.323	1.824 ± 0.438	1.354 ± 0.066
Heart	0.779 ± 0.078	0.241 ± 0.013	0.204 ± 0.025	0.147 ± 0.009
Spleen	0.999 ± 0.218	1.840 ± 0.618	2.172 ± 0.391	2.631 ± 0.199
Stomach	0.206 ± 0.044	0.469 ± 0.438	0.311 ± 0.153	0.571 ± 0.389
Intestine	0.977 ± 0.313	4.242 ± 0.113	3.661 ± 1.491	0.196 ± 0.055
Colon	0.108 ± 0.031	0.044 ± 0.003	1.566 ± 1.539	1.809 ± 0.976
Testes	0.054 ± 0.004	0.033 ± 0.001	0.030 ± 0.005	0.022 ± 0.003
Skin	0.290 ± 0.039	0.196 ± 0.017	0.169 ± 0.029	0.100 ± 0.010
Muscle	0.157 ± 0.004	0.056 ± 0.007	0.048 ± 0.006	0.037 ± 0.005
Thyroid	0.551 ± 0.040	0.225 ± 0.021	0.189 ± 0.009	0.124 ± 0.037
Brain	0.049 ± 0.003	0.013 ± 0.001	0.014 ± 0.003	0.007 ± 0.001
Fat	0.305 ± 0.060	0.120 ± 0.019	0.105 ± 0.017	0.070 ± 0.017
Femur	0.330 ± 0.036	0.202 ± 0.015	0.218 ± 0.031	0.232 ± 0.037
